# Islet β cell mass in diabetes and how it relates to function, birth, and death

**DOI:** 10.1111/nyas.12031

**Published:** 2013-01-30

**Authors:** Gordon C Weir, Susan Bonner-Weir

**Affiliations:** Section on Islet Cell Biology and Regenerative Medicine, Research Division, Joslin Diabetes Center, Department of Medicine, Harvard Medical SchoolBoston, Massachusetts

**Keywords:** beta cell, islets, diabetes, neogenesis, insulin secretion

## Abstract

In type 1 diabetes (T1D) β cell mass is markedly reduced by autoimmunity. Type 2 diabetes (T2D) results from inadequate β cell mass and function that can no longer compensate for insulin resistance. The reduction of β cell mass in T2D may result from increased cell death and/or inadequate birth through replication and neogenesis. Reduction in mass allows glucose levels to rise, which places β cells in an unfamiliar hyperglycemic environment, leading to marked changes in their phenotype and a dramatic loss of glucose-stimulated insulin secretion (GSIS), which worsens as glucose levels climb. Toxic effects of glucose on β cells (glucotoxicity) appear to be the culprit. This dysfunctional insulin secretion can be reversed when glucose levels are lowered by treatment, a finding with therapeutic significance. Restoration of β cell mass in both types of diabetes could be accomplished by either β cell regeneration or transplantation. Learning more about the relationships between β cell mass, turnover, and function and finding ways to restore β cell mass are among the most urgent priorities for diabetes research.

## Introduction

While type 1 diabetes (T1D) clearly results from a loss of β cells, there were decades of uncertainty about the contribution of β cell failure to type 2 diabetes (T2D). In the first half of the 20th century it was generally assumed that β cell failure was important for all diabetes. In the 1960s, however, the development of the insulin radioimmunoassay led to the finding that plasma insulin levels were often high in T2D, leading many to assume that the pathogenesis could be explained by insulin resistance alone. However, over the past 15 years there has been general agreement that β cell inadequacy is a fundamental part of T2D. The growing incidence of T2D is driven by insulin resistance, which is likely the result of a typical Western lifestyle characterized by obesity and minimal exercise. A key point is that most people with insulin resistance will never develop T2D: diabetes only results if their β cells fail to provide sufficient insulin. This review will focus upon the relationship between β cell mass and function in diabetes, and the complicated issues of β cell birth, death, and replenishment.

## β cell mass

In a normal adult human pancreas, β cell mass is approximately 2% of pancreatic weight. About one million islets are scattered throughout the pancreas, which translates to roughly one billion β cells. Pancreases typically weigh between 60 g and 100 g, so β cell mass is somewhere near 1–2 g.

**Figure 1:**
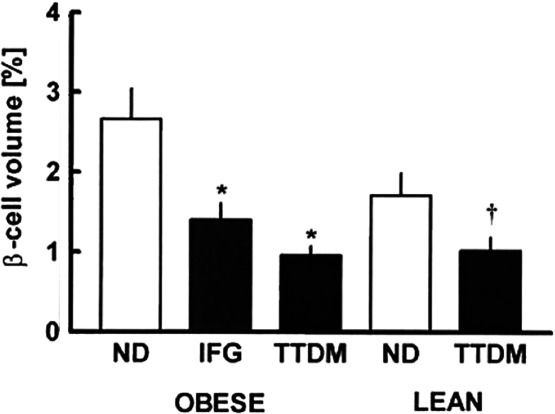
β cell relative volume in autopsy pancreases from individuals who were nondiabetic (ND) had impaired fasting glucose (IFTG) or had type 2 diabetes mellitus (TTDM). Figure taken from Ref. 2, with permission from the American Diabetes Association.

In 1955 Maclean and Ogilvie^1^ reported, in a careful study on autopsy pancreases using histochemical staining, that β cell mass in older people with diabetes (presumably mostly T2D) was about 50% of controls, although this finding was largely ignored. It was also found that α cell mass was not altered. Similar estimates of β cell mass were found in a few small studies, and then in 2003 a study led by Butler and Butler reported similar findings using immunostaining in autopsy pancreases from better-characterized subjects.^2^ There was considerable scatter in the data, but in both lean and obese individuals the relative β cell volume, a surrogate for mass, was about 30–70% of nondiabetic controls. Importantly, in subjects with impaired fasting glucose levels, the β cell mass was also reduced, but less so, to about 60% of normal (Fig. 1). Thorough studies from Korea and Belgium reported similar reductions in β cell mass in T2D.^3^,^4^ Another finding important for understanding T2D is that obese nondiabetic subjects in three studies had only a 20–40% increase in β cell mass compared to lean controls.^2^,^4^,^5^ This only modest increase was surprising, as the increase of β cell mass in insulin resistant mice is proportionally much higher.^6^

In T1D, β cells are decimated by autoimmunity and the process usually occurs over several years. There are few pathological studies and β cell imaging has not advanced enough to be helpful, so our assumptions are largely extrapolated from C-peptide secretion and insulin requirements. Because some individuals with new-onset T1D have remissions that can last for several months, it is thought that β cell mass at diagnosis might be as high as 50% of normal in some individuals.^7^ We know from the Diabetes Control and Complications Trial (DCCT) that C-peptide levels can be substantial in some subjects soon after diagnosis but then fall to negligible levels in just a few years.^8^ A new remarkable finding is that β cells may never be completely wiped out in T1D. The Joslin Medalist Study includes subjects who have had T1D for over 50 years.^9^ In recent years a number of them have died and donated their pancreases for study. At this writing, β cells identified by insulin immunostaining have been found in all 28 subjects (S. Bonner-Weir, unpublished). It also appears that those few with more residual C-peptide have more β cells. The presence of these β cells raises important questions as to whether β cells have special characteristics that resist destruction or whether new β cells are continually formed by replication of existing β cells and/or by neogenesis and then destroyed by persisting autoimmunity.

## The quest to measure β cell mass with imaging

Accurate measurement of β cell mass in living subjects would greatly enhance our understanding of the pathogenesis of both T1D and T2D, as well as the monogenic forms of diabetes, making it a major research priority. For both types it would be of great value to follow the dynamics of the decline in β cell mass because it might be possible to link these to pathogenetic mechanisms. In T1D this could be enormously helpful in evaluating the efficacy of various immune interventions that are being tested in subjects with prediabetes and new-onset diabetes. In both types of diabetes we would very much like to understand the effects of medication and glycemic control on β cell mass.

Unfortunately, it has proved very difficult to develop any methods that come close to being accurate. There was great interest in the type 2 vesicular monoamine transporter (VMAT2), which is expressed by β cells, can be labeled with [^11^C]-dihydrotetrabenazine, and then measured with PET scanning. Unfortunately, in human subjects this appears to be insufficiently specific.^10^ Now the GLP-1 receptor, which may be specific enough for β cells, is receiving much attention.^11^ Another approach is to tag inflammation in the pancreas of T1D, which correlates with the activity of autoimmunity; the first published results in humans look promising.^12^ Most of the approaches to imaging β cells over the past several years have been recently reviewed.^13^ One caveat is that to be useful a method will probably need to accurately measure differences in β cell mass in the range of only 5%, because such small changes are likely to make a difference in glycemic control.

## Normal β cell function and maintenance of mass

β cells have a remarkable ability to make and store large quantities of insulin and then secrete this product with exquisite timing and precision. Glucose is the dominant factor controlling β cell function and survival, and a unique glucose recognition mechanism allows control by extracellular concentrations of glucose that usually range between 4 mM and 8 mM (70–140 mg/dL). This is accomplished by wide-open GLUT2 glucose transporters in rodents, although probably more by GLUT1 in humans.^14^,^15^ These transporters allow the extracellular and intracellular glucose concentrations to be essentially the same. The key glucose signaling for insulin secretion is done by glucose metabolism, the rate of which is controlled by glucose phosphorylation by glucokinase, with a *K*_m_ of about 7 mM.^16^,^17^ Glucose metabolism at the mitochondrial level turns on secretion via the reasonably well-understood K^+^-ATP-dependent pathway and the less well-understood K^+^-ATP independent pathway.^18^ The β cell also responds to signals from the brain via both branches of the autonomic nervous system^19^ and the incretin hormones from the intestine, gastrointestinal insulinotropic peptide (GIP), and glucagon-like peptide 1 (GLP-1).^20^

The β cell provides very efficient control of glucose levels with eating as secreted insulin starts activating the liver even before blood glucose levels rise, due to vagal stimulation during the cephalic phase of insulin secretion^17^ and secretion of incretin hormones. As insulin secretion rises, glucagon secretion is suppressed, allowing this increased insulin/glucagon ratio to enhance hepatic glucose uptake at just the right time.^21^ Protection against hypoglycemia is particularly dependent upon rapid shutdown of insulin secretion by falling glucose levels.^22^ Thus glucose and other factors can turn on insulin secretion within minutes and, when removed, shut it off as quickly.

### Oscillations of insulin secretion

Insulin secretion exhibits oscillation with a periodicity of about five minutes,^23^,^24^ which appears to be coordinated by intrapanceatic neural connections.^25^ Because these oscillations are so prominent in the portal vein, they likely have important influence on insulin's effects upon the liver. Disruption of these oscillations has been found in T2D,^26^ and it could have a negative influence on hepatic insulin action.

### β cell hypertrophy and atrophy

Glucose appears to be dominant among the factors influencing the maintenance of β cell mass. Changes in glucose levels seem to drive the major determinants of β cell mass, hyperplasia, hypertrophy, and atrophy. Chronic hypoglycemia, which occurs with insulin-secreting tumors, leads to marked β cell atrophy,^27^ whereas chronic hyperglycemia can lead to β cell hyperplasia and hypertrophy.^28^,^29^ Using partial pancreatectomy to create a model of hyperglycemia in rats, we found that individual β cell size increased by 85%.^29^ Increases in glucose levels stimulate replication within a day,^30^ and over time lead to hyperplasia. Glucose stimulation of β cell replication has also been found in an *in vivo* model of glucose infusion in mice^31^ and an *in vivo* model of human islet transplantation.^32^

### Compensatory β cell response to insulin resistance when blood glucose levels are normal

There has been considerable debate about how β cell secretion and mass can be augmented in insulin resistant states when increases in glucose levels cannot be determined. We favor the view that because glucose is such a dominant determinant of β cell function and growth, these changes are mainly controlled by extremely efficient glucose feedback on β cells.^6^,^33^,^34^ There may be subtle changes in glucose levels that make a difference and there is evidence of increased activity of glucokinase,^35^ which means that a β cell can be more responsive at lower glucose concentrations. There is much interest in the possibility that some important signals are produced by the liver because of the impressive β cell compensation found with knockout of hepatic insulin receptors in mice.^34^ The search continues.

## Dysfunctional insulin secretion as diabetes develops

**Figure 2:**
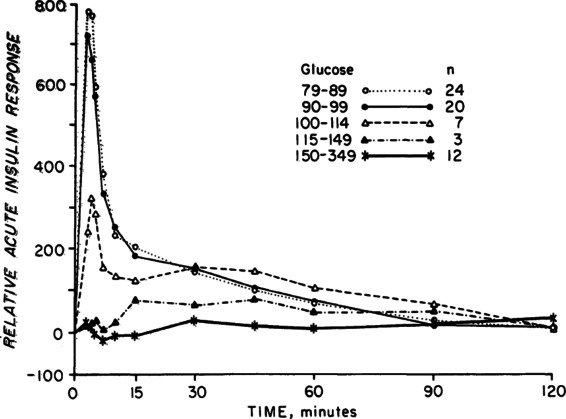
Increments of acute GSIS in subjects with increasing fasting plasma glucose levels. Figure taken from Ref. 36, with permission from the Endocrine Society.

When glucose levels chronically rise to levels only modestly higher than normal, dramatic dysregulation of insulin secretion appears. This was shown most impressively with a simple experiment published over 35 years ago (Fig. 2).^36^ Adult humans with various levels of fasting glycemia received rapid infusions of glucose intravenously to elicit acute glucose-simulated insulin secretion (GSIS). When the fasting glucose was normal at 4.5–5.6 mM (80–100 mg/dL) a large spike of insulin secretion appeared within just a few minutes. However, the magnitude of GSIS was much lower when glucose levels rose above 5.6 mM and by the time they reached 6.4 mM (115 mg/dL), a level in the range of impaired fasting glucose (IFG), acute GSIS, a prediabetic state equated with first-phase insulin secretion, was completely obliterated. Nonetheless, the β cells functioned well enough to maintain the prediabetic state because they can respond to more prolonged glucose stimulation with second phase release^37^ and to acute stimulation by incretin signals such as GLP-1, as well as amino acids. These findings have now been reproduced in multiple human and animal studies.

**Figure 3:**
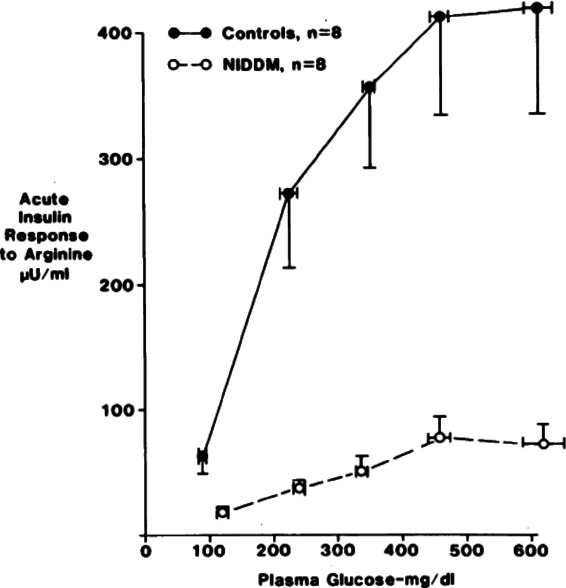
Subjects with noninsulin-dependent diabetes (NIDDM, T2D) and control subjects whose glucose levels were increased with glucose infusions followed by acute stimulation of insulin secretion with intravenous arginine. Figure taken from Ref. 38, with permission from the Endocrine Society.

Dysfunction of β cells becomes more serious as the diabetic state worsens and functional mass deteriorates. A given β cell mass puts out less insulin in response to stimuli. In another old study, subjects with and without T2D received maximal β cell stimulation from prolonged infusions of glucose augmented with arginine.^38^ It can be assumed that the β cell mass of these T2D subjects was in the range of 50% of normal, yet their insulin response to this maximal stimulus was only 15% of normal (Fig. 3).

Importantly from a therapeutic perspective, the severe dysfunction induced by the diabetic state can be reversed if glucose levels are brought to normal, as best shown by the full restoration of secretion after bariatric surgery.^39^ It is surprising how little we know about the timing of this restoration. In T2D, partial improvement in GSIS was found after a 20-hour infusion of insulin^40^ and changes after gastric surgery were found weeks to months later. This is an important question because a thorough understanding of the timing and magnitude of the effects of glucotoxity could have therapeutic value.

## The case for the importance of glucotoxicity and lack of importance of lipotoxicity and glucolipotoxicity

While it is clear that the diabetic milieu is responsible for β cell dysfunction, there has been much discussion about the contributions of glucotoxicity, lipotoxicity, and glucolipotoxicity.^41^–^43^ The case for the dominance of glucotoxicity can be made because of the remarkably tight correlation between glucose levels and loss of acute GSIS. Moreover, changes in insulin secretion after several days of glucose infusions in rats resemble those seen in diabetics,^44^ yet such infusions suppress free fatty acid (FFA) levels. Also, similar loss of GSIS can be found when isolated islets are exposed to high-glucose concentrations in culture.^45^,^46^

The case for lipotoxicity is based almost entirely upon *in vitro* exposure of primary β cells and β cell lines to FFAs in culture, especially the saturated FFA palmitate. Not only does palmitate disrupt GSIS and other types of insulin secretion, but also it has become a favorite tool in the study of β cell death. It is not at all clear that the FFA concentrations used for these *in vitro* studies are similar to those seen by β cells *in vivo*. We have little understanding of how FFAs are handled in the interstitial space and the environment of lipid membranes that surround β cells. It has also been postulated that palmitate given *in vitro* to β cells may be converted to the toxic product tripalmitin, which is not expected to be formed *in vivo*.^47^ An apparent shortcoming in the use of FFAs or high glucose concentrations^48^ to study β cell death is that the frequency of death in so many *in vitro* studies is far higher than the frequency seen *in vivo*, which raises concerns about the relevance of some of the mechanisms that have been identified. There are a few *in vivo* studies using infusions of lipid,^49^ but the resultant FFA levels are so high that their clinical relevance must be questioned.

Data obtained from *in vivo* studies on lipotoxicity in humans and animal models are also unconvincing. For example, FFA levels are high in obesity, which is associated with exuberant GSIS. The increase of glucose levels in prediabetes correlates very precisely with GSIS dysfunction,^36^ but a similar correlation with FFA has yet to be demonstrated. It has also been postulated that increased lipid stores in the form of droplets cause toxicity, but a correlation between lipid stores and secretory dysfunction in β cells has yet to be shown.^50^ Moreover, β cell secretory dysfunction can be found in db/db mice independent of plasma lipid levels.^51^ The obese Zucker diabetic fatty (ZDF) rat model of diabetes has been proposed as an example of lipotoxicity because the late stage islets contain large quantities of lipid as diabetes worsens.^52^ The problem is that it has never been shown that most of this lipid is located in β cells rather than the fibroblasts and adipocytes that can be seen in these anatomically distorted islets.

Proponents of lipotoxicity found that *in vitro* exposure of β cells to FFAs and high glucose was even more damaging, which led to the widely used term glucolipotoxicity. The problem remains that FFAs may be toxic only in an artificial *in vitro* environment. In conclusion, while lipotoxicity is a major issue for the pathogenesis of insulin resistance in various insulin target tissues, evidence that lipotoxicity and the lipid part of glucolipotoxicity contribute to β cell secretory dysfunction or death in human diabetes or animal models is presently weak to nonexistent

## Reduction of insulin content and abnormal proinsulin secretion

β cells contain remarkable amounts of insulin, about 10% of the total protein content with an average β cell, about 20 pg of insulin. Insulin synthesis is balanced by insulin secretion and by the autophagic destruction of secretory granules. Because insulin mRNA has a long half-life and stable concentration,^53^ the fine-tuning of replenishment after meals seems mostly driven by glucose-stimulated translation. When β cells are exposed to the diabetic state, they become degranulated such that in some experimental systems the content per β cell mass can be reduced to lower than 5% of normal.^54^ Data from a variety of sources indicate that insulin depletion is much more severe with high rather than modest elevations in blood glucose. While secreted insulin accounts for some of this depletion, the reduction of insulin synthesis must be a critical component.^55^

Of secreted insulin immunoreactivity from β cells, about 2–4% is proinsulin, while circulating proinsulin in blood can be 10–40% of the total due to its longer half-life. The proinsulin to insulin ratio in blood is increased in T2D and to a lesser extent IGT and has been used as a marker of β cell dysfunction. This proportional change is thought to result from depletion of mature granules resulting in increased secretion of the incompletely processed contents of immature granules.^56^

## Five stages of diabetes; clinical consequences of β cell dysfunction induced by prediabetic and diabetic states

To understand both T1D and T2D, it is helpful to conceptualize five stages in their progression (Table 1).^57^ Stage 1 is successful compensation for a modest fall in β cell mass in T1D or for insulin resistance in T2D. The β cells secrete more insulin to maintain a normoglycemic environment, which in turn allows acute GSIS because β cells are not exposed to glucotoxicity. With stage 2, β cell mass drops a little more and/or insulin resistance worsens, such that glucose levels rise to levels associated with a marked loss of acute GSIS, which we postulate is caused by glucotoxicity. Stage 2 is equivalent to impaired glucose tolerance (IGT) or IFG, which is stable because of maintenance of meaningful insulin secretion, such that only about 5% of individuals with IGT progress to T2D per year.^58^ Stage 3 is an unstable phase in which increasing glucotoxicity produces more β cell dysfunction and insulin resistance, leading to a rapid rise in glucose levels to stage 4, with more serious hyperglycemia in the range of 9–17 mM (160–300 mg/dL). This relatively rapid deterioration in control may not be caused by a fall in β cell mass but instead by increased insulin resistance from stress and overeating, for example. Evidence supporting the existence of stages 2–4 in humans was recently reported by the Whitehall study group.^59^ Stage 5 has more severe β cell depletion as found in T1D.

**Table 1:** Five stages of diabetes^57^

**Stage 1**: Normal blood glucose levels: compensation for insulin resistance with increased β cell insulin secretion and mass. With early autoimmune destruction of T1D, increased insulin secretion compensates for decreased β cell mass.
**Stage 2**: Stable adaptation: IGT, IFG, pre-T1D, islet transplantation. Inadequate β cell mass allows modest hyperglycemia, which leads to dysfunctional GSIS from glucotoxicity.
**Stage 3**: Unstable decompensation: T2D and early T1D; worsening glucotoxicity for β cells and insulin target tissues causes a rapid rise in blood glucose levels.
**Stage 4**: Stable decompensation: higher blood glucose levels in T2D and early T1D, but enough insulin secretion to control ketosis.
**Stage 5**: Decompensation: T1D; classic symptoms of diabetes and ketosis; severe reduction in β cell mass.

These concepts are therapeutically significant for T2D because aggressive treatment of subjects in stage 4 may be able to reduce glucose levels to the more stable stage 2 of IGT, or to an even better stage 1. There are already suggestions that aggressive early treatment of newly diagnosed patients with T2D with stage 4 glucose levels may lead to lasting improvement in control.^60^ These stages are also important for T1D because the β cell appears to go through the same development of dysfunctional insulin secretion as autoimmunity reduces β cell mass.^61^ For example, subjects with new-onset T1D may present with high glucose levels and then have a remission that is either spontaneous or induced by immunosuppression.^7^ During remission C-peptide levels might be considerably higher than when patients initially presented with hyperglycemia.^62^ Despite hopes that this could be explained by β cell regeneration, relief from glucotoxicity leading to improved β cell function seems like a better explanation.

## β cell birth and death

To understand changes in β cell mass one needs to understand β cell turnover, which is better understood in rodents than in humans. Birth can result from replication of preexisting β cells or from neogenesis, the formation of new islets thought to originate from cells in the pancreatic duct epithelium. Evidence is accumulating that some cells with duct identification markers can serve as multipotent precursor cells.^63^,^64^ There has been debate and even controversy about the relative roles of replication versus neogenesis. Replication has been shown to be particularly important in adult β cell expansion^65^ and for regeneration after β cell depletion from diphtheria toxin in mice.^66^ In fetal life new islets are formed from cord- or duct-like structures, and it is logical to think that postnatal neogenesis is largely a recapitulation of this process. In spite of discordant lineage tracing results in mice,^63^ it is now better appreciated that substantial neogenesis occurs during the neonatal period^67^,^68^ and in response to some forms of injury.^63^,^69^,^70^

In humans the majority of β cell expansion occurs in early childhood with probably a greater contribution from replication than from neogenesis.^71^ In adults, some studies find no evidence of any β cell replication;^72^,^73^ however these findings are not straightforward. For example, when adult human islets are transplanted into immuno-incompetent mice, they always have Ki67^+^ β cells in the range of 0.2–0.7%.^74^ Perhaps there is something about the mouse *in vivo* environment that turns on quiescent cells. It may be significant that almost all studies have used pancreases obtained from autopsies or cadaver donors, which raises questions as to whether the warm and cold ischemia of these conditions dampens the Ki67 expression. This is supported by the finding that in pancreatic samples obtained at surgery Ki67 positivity was found to be in the range of 0.5%.^75^

**Figure 4:**
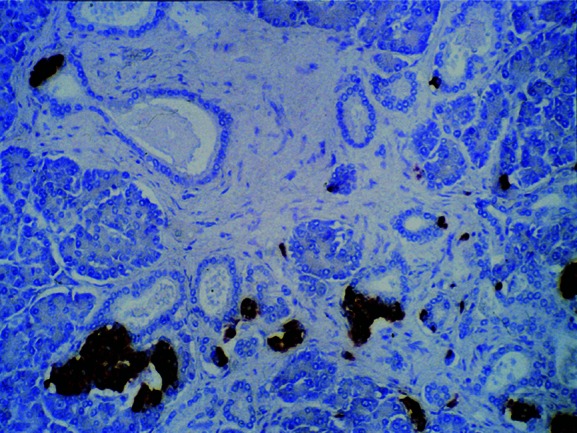
Section of pancreas (20× magnification) stained for insulin. Numerous pancreatic ducts are shown, with insulin-positive cells present in and next to duct wall, suggesting new islet formation from exocrine ducts (neogenesis). Figure taken from Ref. 2, with permission from the American Diabetes Association.

There appears to be a low rate of β cell death, as judged by such techniques as TUNEL.^2^ Because β cell mass is reasonably well maintained in nondiabetics, it makes sense that there would be some low level of turnover, with birth balancing death. Determining the contribution of neogenesis in humans is very difficult, but the presence of small clusters of insulin-containing cells within, protruding from, and near ducts (Fig. 4), and the discovery of cells double stained for insulin and the duct marker cytokeratin, are consistent with some level of neogeneic activity.^63^ The severe hypoglycemia sometimes seen after bariatric surgery is associated with pancreatic pathology consistent with neogenesis.^76^ There is speculation that this is driven by the very high plasma levels of GLP-1 seen in these patients.^77^ Regardless of how β cells are born, they are certainly long-lived; this is supported by the finding that a very high percentage of β cells in adult humans contain lipofuscin, which takes a long time to accumulate.^78^

The possible significance of a low rate of turnover should not be underestimated. If 0.5% of β cells are Ki67^+^ and Ki67 positivity lasts for 12 hours, β cell mass could more than double in less than a year. The changes in mass will of course also be influenced by the variables of death (from apoptosis and necrosis) and neogenesis, which are even harder to quantify.

## What causes the reduction of β cell mass in T2D?

Many conclude that the reduced β cell mass in T2D reflects an increased rate of β cell death, but while this must often be true, there may also be a β cell birth problem. For example, T2D may result from an intrauterine growth disturbance or genetic and environmental forces that slow β cell replication or neogenesis.

There is a lot of circumstantial evidence that the prediabetic/diabetic state, or more specifically glucotoxicity, leads to an increased rate of β cell death from either apoptosis or necrosis. However, overwork in the absence of increases in glucose levels seems to be associated with reduced β cell mass. The observation by the Butler group that individuals with IFG (stage 2) have, on average, a 40% reduction in β cell mass (Fig. 1)^2^ suggests that something deleterious occurs during the compensatory stage 1. We know that glucose can stimulate replication,^30^ and we can argue that it still has an effect in insulin resistant states when glucose levels appear to be normal, as in stage 1. We hypothesize that during this stage of compensation there is increased replication of β cells, and suspect that there is some limitation in the capacity for regeneration through glucose-driven replication leading to slowing of birth. Thus, during this time of overwork a limitation in the birth of new cells could lead to an older population of β cells that has a higher rate of cell death, which could contribute to the impressive reduction in β cell mass found in the earliest stages of diabetes of IGT and IFG, and could help explain the increased incidence of apoptosis later in the disease.

While investigators may advocate for the dominance of their favorite mechanism, it may be best to take the position that several mechanisms might contribute.

### Oxidative stress

There has been great interest in the possibility that glucotoxicity leads to oxidative stress, which then causes secretory dysfunction and an increased rate of β cell death.^79^ Certainly glucose stimulation leads to an increase in reactive oxygen species (ROS), but this is not necessarily bad. There is interesting research underway to sort out the contributions of ROS to important β cell signaling mechanisms that are part of normal β cell function and to β cell dysfunction and death.^80^ This has led to the hope that antioxidant treatment might slow the adverse processes.^81^ Indeed, the administration of *N*-acetyl-cysteine has been found to slow the adverse effects of ROS on the progression of diabetes in ZDF rats.^82^

### Amyloid

The first description of amyloid deposits, called hyaline at the time, in the islets of people with diabetes was reported in 1900.^83^ It took until 1987 to determine that this amyloid was formed from the β cell secretory product islet associated polypeptide (IAPP),^84^ which in humans and a few other species can form the β-pleated sheets that make up amyloid. The question as to whether amyloid deposits are just innocent bystanders or whether its formation somehow contributes to cell death appears to have been answered. Examination of pancreases from subjects with T2D indicated that islets with large amyloid deposits often have fewer β cells.^85^ When human IAPP, which can form amyloid unlike rodent IAPP, is overexpressed in β cells of mice or rats, the result is worsening of diabetes associated with more amyloid deposition.^86^–^88^ It now appears that the toxicity is exerted not by the extracellular deposits but by small fibrils or toxic oligomers that can damage cell membranes.^89^,^90^ There is increasing evidence that these can cause damage while inside the β cell; they may when outside as well. It is still puzzling that amyloid deposits are seen in islets of T2D and in insulinomas but are far less common in obesity, in spite of the increased secretion of both insulin and IAPP.

### Endoplasmic reticulum stress

The potential contribution of endoplasmic reticulum (ER) stress to β cell apoptosis in T2D has received much attention in the past few years.^91^–^93^ ER stress is part of the unfolded protein response (UPR). This UPR is turned on when the cell has problems with the complex process of folding newly synthesized proteins. To protect the cells, it recruits chaperone proteins that help with folding, accelerates the degradation of unfolded proteins, and inhibits the synthesis of new proteins. While the UPR has important benefits for function and survival, a fully turned on stress response activates apoptotic pathways through such mediators as C/EBP homologous protein (CHOP) and c-Jun N-terminal kinase (JNK). A study of gene expression in human β cells transplanted into mice showed that these cells, in a transplant site exposed to mild hyperglycemia, had an activated UPR but reduced expression of JNK and CHOP,^94^ suggesting that ER stress is more helpful than harmful in that situation. In our study of pancreases from cadaver donors with T2D, gene expression was performed on laser-captured β cell-enriched tissue with little change in ER stress genes, although there were a few changes suggesting that parts of the UPR were activated.^95^

Butler's group stained for CHOP in pancreases from subjects with T2D, and from lean and obese subjects without diabetes.^96^ The β cells of both the T2D and obese individuals had cytoplasmic CHOP staining in about 15–20% of their β cells. However, nuclear staining for CHOP was seen in less than 1% of the β cell from T2D, and nuclear staining in the obese subjects was even lower. This is consistent with the view that only small numbers of β cells in T2D are dying at any one time, but that a larger number are showing signs of stress or fragility. Thus, the few cells with nuclear CHOP could be on their way to death while those with cytoplasmic CHOP may be more vulnerable but still functional. Likewise, it may be that in T2D many β cells have some UPR activation, which protects them, but in a more fragile, perhaps older, population of β cells, the proapoptotic ER stress pathways are more active.

### β cell dedifferentiation

Gene expression in β cells exposed to hyperglycemia can be studied after surgical reduction of β cell mass in rats with partial pancreatectomy.^29^,^50^,^97^–^99^ After various periods of time, the β cells in isolated islets and in tissue sections from the remnant pancreas can be studied. Impressive changes in gene expression were found in β cells exposed to either very modest or severe hyperglycemia. These changes included reduced expression of several key β cell transcription factors, changes related to glucose metabolism, and upregulation of genes related to stress. It has been tempting to call this change in β cell phenotype dedifferentiation, but it is not possible to say that these cells reverted to a phenotype that occurred earlier in development. While it makes sense that some of these changes could have a deleterious effect on secretion, little can be said about how they might be related to a fall in β cell mass.

A new look at phenotypic change was taken by the Accili group, which obtained data from mice lacking FoxO1 and suggested the novel hypothesis that in T2D β cells are dedifferentiated to a state of immaturity whereby they express neurogenin3, Oct4, Nanog, and L-Myc and no longer make insulin.^100^ Some of these cells even adopted an α cell fate. The argument was made that restoration of differentiation in these cells may be a therapeutic path to increase β-cell mass in T2D. This work will no doubt stimulate new research, but at this early stage questions must be raised as to whether a FoxO1 deficiency model is relevant to T2D. Certainly investigators will take a closer look at human pancreatic T2D islets for immature cells. The possible conversion of β cells to α cells is interesting but α cell mass in human T2D has not been found to be increased in two thorough studies.^1^,^101^

## Need to better characterize β cells to understand pathogenesis

Most of our understanding comes from studying large numbers of islet cells, but much more could be learned about pathogenesis if we could learn more about the heterogeneity of β cell death. For example, why is it that only one in many hundred cells is chosen to enter the cell cycle and replicate? Likewise, what is special about the one cell of many that dies? We know from many studies there is considerable heterogeneity for the capacity to secrete insulin. A human β cell may have a lifetime of a several years, so there must be differences among cells that are new, in the early stages of maturation, in midlife, in the stage of postmitotic senescence, when they are old and fragile, and finally when they are dying. Also, what are the differences between a new β cell formed by neogenesis in a fetus versus one that formed during adulthood? And, what are the differences between β cells formed from neogenesis compared with those produced from the replication of existing cells? How important is topical location, such as the islet center or next to the mantle of non-β cells? Could there be different fetal pathways of development the lead to the generation of different kinds of β cells? What might the differences be between β cells in the dorsal versus the ventral lobes of the pancreas? What happens to β cells in islets that are less active and have less blood flow?^102^ One might guess that there is temporal cycling of β cells that allows them to rest until needed. Surely the differences described above are accompanied by changes in gene and protein expression, so there must be ways to find markers for many of the different β cell types. Flow cytometry and histochemical staining may be the most promising ways to isolate these different cell types for study. Even if only some of these possibilities are realized, it is exciting to think of how much might be learned about how β cells function, are born, and die.

## Restoration of β cell mass in diabetes

We know that restoration of β cell mass can normalize blood glucose levels in both T1D and T2D. The proof-of-principle has been established with islet transplantation in T1D^103^ and pancreas transplantation for T2D.^104^ Unfortunately current approaches require cadaver pancreases and immunosuppression, which greatly limits the number of diabetics that can be treated. To make β cell replacement therapy more accessible, we need an abundant supply of well-characterized β cells and must find safe ways to prevent autoimmune and allogeneic killing of the newly provided β cells. Possibilities for replacement include cellular transplantation and regeneration of pancreatic endocrine cells.

For a source of transplantable cells, islet cells derived from human embryonic stem cells or induced pluripotent stem (IPS) cells are particularly attractive because there have been impressive scientific advances in recent years.^105^–^107^ Another hope is to find ways to expand human pancreatic cells *in vitro* so they can be transplanted. There are a few encouraging studies suggesting that β cells or pancreatic duct cells can be directed to a mesenchymal phenotype, allowing expansion and then redifferentiation to islet cells.^108^,^109^ The possibility of stimulating β cell replication has also been receiving considerable attention, with the identification of new compounds that can stimulate division^110^,^111^ and the elucidation of mechanisms that might be exploited in the future.^112^–^116^ Another approach is to create a β cell line with genetic engineering that can be redifferentiated before transplantation, but safety concerns are likely to remain.^117^ Finally, the possibility of xenotransplantation with porcine islets from adult or perinatal pigs continues to be explored.^118^,^119^

Finding ways to limit autoimmunity and allorejection has been a daunting endeavor. With stem cells, it should be possible to use lines that will allow favorable human leukocyte antigen (HLA) matching. Transplantation of islet cells derived from iPS cells generated from people with T2D should face no immune attack, but those derived from subjects with T1D should face a strong autoimmune reaction. There are many approaches to control allo- and autoreactivity that are beyond the scope of this review, but the possibility of protecting cells with immunobarrier membranes is receiving renewed attention.^120^ It seems likely that the first transplants of islet cells derived from stem cells will employ this protection method. The general approach is to use either macroencapsulation, in which many islets are contained within a device often called a bioartificial pancreas, or microencapsulation, in which a small number of islets or cell aggregates are contained in small hydrogel capsules that could be 400–1500 μm in diameter. Islet cells can also be protected with conformal coating using polyethylene glycol (PEG) or similar materials.^121^

Finally, there is the dream of stimulating regeneration of β cells in the pancreas.^122^ Drugs might be developed that could stimulate β cell replication or neogenesis. Another possibility is that the exocrine pancreas, or possibly even cells in the liver, could be reprogrammed to become β cells.^123^ In a striking example of reprogramming, a single injection of adenoviruses carrying genes for three transcription factors important for β cell development (Pdx1, neurogenin3, and MafA) into a mouse pancreas can produce large numbers of cells with β cell characteristics that can store and release enough insulin to reverse the diabetic state.^124^

## Summary

Reduction of β cell mass is fundamental to the pathogenesis of both T1D and T2D and leads to severe dysfunction of insulin secretion. These two abnormalities lead to the concept of the reduced β cell functional mass. We need to know more about how reduced mass results from unbalanced β cell turnover, and understand how this leads to secretory dysfunction. Diabetes can be prevented or reversed by inhibiting the fall of β cell mass or by restoring mass through regeneration or transplantation. These issues are among the highest priorities for the field.
